# Correction

**DOI:** 10.1080/23320885.2026.2743460

**Published:** 2026-10-07

**Article Title:** Giant thigh lipomas: from a case report to a guide for the surgical approach

**Authors:** De Maria, F., Marra, C., Blessent, C. G. F., Starnoni, M., Pappalardo, M., and De Santis, G.

**Journal:** *Case Reports in Plastic Surgery and Hand Surgery*

**Bibliometrics:** Volume 11, Number 01, pages 1–4

**DOI:** 10.1080/23320885.2024.2335275

When the article was initially published online, the abstract contained an error.

Please find the revised abstract below:

**Background:** Giant lipomas of the thigh are uncommon benign soft tissue tumors that may cause functional impairment and, in rare cases, compression of major neurovascular structures. Their surgical management requires careful preoperative assessment and planning because of the potential risk of vascular complications and postoperative wound complications.

**Case presentation:** We report the case of a 56-year-old woman with a progressively enlarging mass of the left thigh, associated with pain, functional impairment, and cutaneous edema. Doppler ultrasound and magnetic resonance imaging demonstrated a large lipomatous mass measuring 18 × 10 × 8 cm, located in the femoral fossa and causing compression of the femoral vein without venous obstruction. The patient underwent surgical excision of the mass. A well-demarcated and encapsulated tumor was identified and carefully dissected from the surrounding structures, resulting in decompression of the femoral vein. The tumor weighed 1705 g. Histopathological examination confirmed a benign lipoma. The postoperative course was uneventful, and the patient was discharged after 2 days. At 6-month follow-up, the patient reported complete satisfaction.

**Conclusion:** Giant thigh lipomas require careful preoperative radiological evaluation and surgical planning, particularly when located in proximity to major vascular structures. Complete and cautious excision, together with appropriate postoperative management, can allow safe treatment and minimize complications. This case emphasizes the importance of assessing possible vascular compression before surgery and of performing giant lipoma excision in an appropriate surgical setting.

This has been updated and republished online.

